# Idiopathic premature ventricular contractions and ventricular tachycardias originating from the vicinity of tricuspid annulus: Results of radiofrequency catheter ablation in thirty-five patients

**DOI:** 10.1186/1471-2261-12-32

**Published:** 2012-07-10

**Authors:** Li Yue-Chun, Zhang Wen-Wu, Zhou Na-Dan, Zhang Teng, Wang Pin-Xiao, Ge Bei, Li Jia, Ji Kang-Ting, Lin Jia-Feng

**Affiliations:** 1 Department of Cardiology, Second Affiliated Hospital of Wenzhou Medical College, 109 Xueyuan Road, Wenzhou, Zhejiang, China

## Abstract

**Background:**

In recent years, catheter ablation has increasingly been used for ablation of idiopathic premature ventricular complexes (PVCs) or ventricular tachycardias (IVTs). However, the mapping and catheter ablation of the arrhythmias originating from the vicinity of tricuspid annulus (TA) may not be fully understood. This study aimed to investigate electrophysiologic characteristics and effects of radiofrequency catheter ablation (RFCA) for patients with symptomatic PVCs and IVTs originating from the vicinity of TA.

**Methods:**

Characteristics of body surface electrocardiogram (ECG) and electrophysiologic recordings were analyzed in 35 patients with symptomatic PVCs/ IVTs originating from the vicinity of TA. RFCA was performed using pace mapping and activation mapping.

**Results:**

Among the 35 patients with PVCs/IVTs arising from the vicinity of TA, complete elimination of PVCs/IVTs could be achieved by RFCA in 32 patients (success rate 91.43%) during a median follow-up period of 21 months. PVCs/IVTs originating from the vicinity of TA had distinctive ECG characteristics that were useful for identifying the precise origin. An rS pattern was recorded in lead V1 in 93.1% of patients with PVCs/IVTs from the free wall of TA, vs 16.7% of patients with PVCs/IVTs from the septal TA, whereas a QS pattern in lead V1 occurred in 83.3% of patients with PVCs/IVTs from the septal TA vs 6.9% of patients with PVCs from the free wall of the TA. The precordial R wave transition occurred by lead V3 or earlier in all patients with PVCs/IVTs originating from the septal portion of the TA, as compared to transition beyond V3 in all patients with PVCs/IVTs from the free wall of the TA.

**Conclusions:**

RFCA is an effective curative therapy for symptomatic PVCs/IVTs originating from the vicinity of TA. There are specific characteristics in ECG and the ablation site could be located by ECG analysis.

## Background

Idiopathic ventricular arrhythmias(VAs), including premature ventricular complexes (PVCs) and idiopathic ventricular tachycardias (IVTs), are the most common arrhythmias observed in patients without structural heart disease [1]. In recent years, radiofrequency catheter ablation (RFCA) has proven to be a safe and successful therapy for the arrhythmias [2-6]. PVCs/IVTs mainly originate from the right ventricular outflow tract, with a small part of them originating from the left ventricular outflow tract [7-9]. Some uncommon sites of idiopathic VA origins have been revealed [10,11], and the tricuspid annulus (TA) may be defined as one of those [12]. However, the mapping and catheter ablation of the arrhythmias originating from the vicinity of TA may not be fully understood. The purpose of this study was to analyze electrophysiological characteristics and the outcome of catheter ablation for such PVCs/IVTs originating from the vicinity of TA.

## Methods

### Study population

From September 2006 to September 2010, a total of 379 patients (148 men and 231 women; age 45.89 ± 19.72 years [mean ± SD]) without structural heart disease presented for catheter ablation for PVCs/IVTs in our hospital. Only patients with idiopathic PVCs/IVTs from the vicinity of TA were enrolled in the present study. All patients had no structural abnormalities by physical examination, routine biochemistry tests, X-ray, color echocardiography examination, exercise electrocardiogram testing, and/or cardiac catheterization with coronary angiography or right ventricular contrast angiography. Before RFCA, a 12-lead ECG was obtained, and 24 hours ambulatory ECG monitoring (Holter) was carried out at least once. The ECG was monitored for 24 hours just before catheter ablation. The selection criteria for the patients with a PVCs/IVTs included the following: (1) frequent or consecutive PVCs occurrence, the average PVC count ≥ 10000 times /24 h; (2) inability of the patient to tolerate PVCs/IVTs or unsuccessful treatment with at least two antiarrhythmic drugs; or patients who did not wish to take long-term antiarrhythmic medications because of specific reasons.

### Ethics approval

Ethical approval was obtained from the Ethics Committee of the Second Affiliated Hospital of Wenzhou Medical College, and all patients gave informed consent before participation in the study.

### Mapping and RFCA

Anti-arrhythmic drugs were withdrawn in all patients at least five half-lives before ablation, with the exception of amiodarone that was withdrawn eight weeks before intervention. Electrophysiologic evaluation and catheter ablation was performed as previously described [4]. Under local anesthesia with 1% lidocaine, a deflectable 7 F quadripolar catheter (Cordis, USA) with a 4 mm distal electrode and an interelectrode spacing of 2-5-2 mm was used for mapping and ablation. A 12 lead surface electrocardiogram was monitored and recorded on a multichannel oscilloscopic recorder. Programmed electrical stimulation was performed from the right ventricular apex at basic drive cycle lengths of 600, 500, and 430 msec, delivering a maximum of three extrastimuli. Pace mapping and endocardial activation mapping were performed. If the clinical arrhythmia did not occur spontaneously and was not induced at baseline, intravenous isoproterenol (2–4 μg/min) was administered to induce arrhythmia. The mapping catheter was first used to localize the His bundle recording. If mapping and ablation was required in the anatomical vicinity of the His bundle, the additional His and coronary sinus quadripolar (Cordis, USA) were introduced.

The target site for RFCA was determined by activation mapping (earliest local activation time preceding the earliest surface QRS by ≥ 25 msec) in patients with frequent PVCs/sustained IVT, and by pace mapping (≥ 11/12–lead concordance of major and minor deflections between the pace map and the clinical PVCs) in those with infrequent arrhythmia. Bipolar pacing was performed at an output just greater than the diastolic threshold from the distal electrode pair (with the distal electrode as the cathode) during sinus rhythm. After the target site was located, ablation was performed using maximum power of 40 W, maximum temperature of 60°C and impedance of 80-140Ωin the temperature-controlled mode. If the PVCs/IVTs terminated within 10 seconds of ablation, or if they increased in frequency within the initial 10 seconds of ablation, additional current was applied for another 60 to 180 seconds. Successful ablation was defined as complete elimination of spontaneous or inducible ventricular arrhythmias. Programmed electrical stimulation was repeated 30 minutes after the last application of radiofrequency energy to confirm the absence of inducible ventricular arrhythmias before removing all catheters and sheaths. If PVCs/IVTs did not terminate within 10 s, the radiofrequency energy application was terminated and another target site was sought.

### Definition of successful ablation

Successful catheter ablation met all three criteria: 1) the absence of spontaneous or induced clinical PVC and ventricular tachycardia, both with or without isoproterenol at the end of the procedure; 2) Absence of PVC or ventricular tachycardia in ECG monitoring over 48 hours without anti-arrhythmic drugs; 3) No recurrence of clinical arrhythmia originating from the vicinity of TA in the absence of anti-arrhythmic drug therapy during follow-up.

### Postablation follow-up

After RFCA, all patients underwent 72-hours ECG monitoring. Holter was carried out one week after RFCA. Patients were not given any antiarrhythmic drugs after RFCA, and underwent color echocardiography and Holter examination three month after RFCA. ECG, echocardiography and 24-hours ECG monitoring were performed whenever the patient had symptoms suggestive of recurrence of VAs. The median follow-up period was 21 months (range 5 ~ 48) months.

### Definition of PVCs/IVTs originating from the vicinity of TA

**Figure 1:**
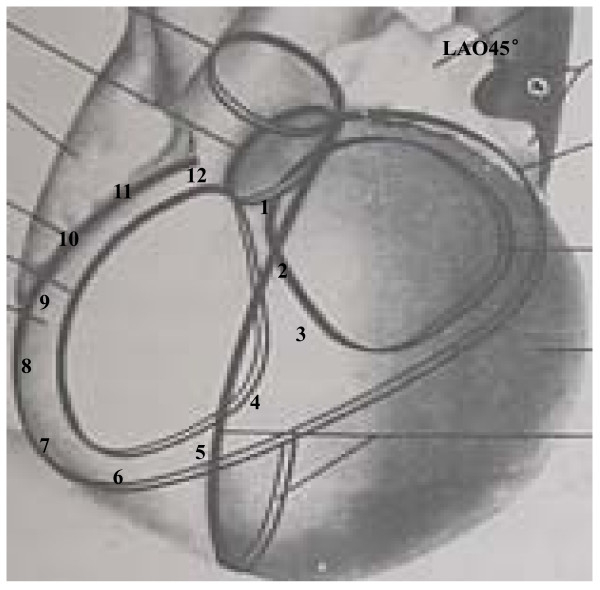
In the left anterior oblique (LAO) projection, the tricuspid annulus was viewed as a clock face, that was divided into 6 portions: anteroseptum (approximately 12 o’clock to 2 o’clock), midseptum (approximately 2 o’clock to 4 o’clock), posteroseptum (approximately 4 o’clock to 6 o’clock position), anterolateral portion (approximately 10 o’clock to 12 o’clock position), midlateral portion (approximately 8 o’clock to 10 o’clock position), posterolateral portion (approximately 6 o’clock to 8 o’clock position).

PVCs/IVTs were considered to originate from the vicinity of TA, based on (1) the characteristic of TA location and motion (when viewed in the right and left anterior oblique fluoroscopic views after successful RFCA); (2) the local endocardial recordings. The ratio of the atrial to ventricular electrograms at the ablation site was <1, and the amplitudes of the atrial and ventricular electrograms were≧0.03 and >0.35 mV at the ablation site, respectively. In the left anterior oblique (LAO) projection, the TA was viewed as a clock face, that was divided into 6 portions (Figure 1): anteroseptum (approximately 12 o’clock to 2 o’clock), midseptum (approximately 2 o’clock to 4 o’clock), posteroseptum (approximately 4 o’clock to 6 o’clock position), anterolateral portion (approximately 10 o’clock to 12 o’clock position), midlateral portion (approximately 8 o’clock to 10 o’clock position), posterolateral portion (approximately 6 o’clock to 8 o’clock position).

### ECG measurements

Twelve-lead electrocardiograms recorded at a paper speed of 25 mm/s were available for all patients with PVCs/IVTs originating from the vicinity of TA during the clinical arrhythmia. The analysis of ECG pattern was focused on the following characteristics: (1) The QRS morphology of the PVCs/IVTs in all 12 leads, (2) the duration of the QRS complex, (3) the site of R-wave transition in the precordial leads, (4) the relationship between the clock’s position of the successful ablation site in LAO and the amplitude of R or S waves in the PVCs/IVTs originating from the right free wall portion of the TA.

### Statistical analysis

All values were expressed as mean value ± standard deviation. The Student’s t-test was used to compare two groups. Analysis of variance (ANOVA) was used when comparisons involved >2 groups, followed by Fisher protected least significant difference test. Bivariate Correlations were performed to determine the relationship between height of R waves or depth of S waves and clock’s position of the origin of PVCs/IVTs in LAO. A value of P < 0.05 was considered significant.

## Results

### Study population

Among patients with PVCs/IVTs, the incidence of TA ventricular arrhythmias was 9.23% (total 35 patients, 22 men and 13 women; mean age 36.91 ± 19.98 years) (Tables 1 and 2). Out of a total of 379 patients treated by RFCA, 351 patients (92.61%) were successfully ablated; the remaining represented ablation failure (Table 1). In the present study, no abnormalities suggestive of arrhythmogenic right ventricular cardiomyopathy were found by ECG and echocardiography and right ventricular contrast angiography in any of the patients with the TA ventricular arrhythmias.

**Table 1:** Tachycardia origin and results of RFCA for idiopathic ventricular arrhythmias

Arrhythmia origin	No.(%)	VT(SVT)	PVCs	Success (%)
Tricuspid annulus	35(9.23)	5(2)	30	32(91.43)
Free wall portion	29(7.65)	5(2)	24	28(96.55)
Septal portion	6(1.58)	0	6	4(66.67)
RVOT	235(62.01)	28(9)	207	224(95.32)
PA	14(3.69)	5(0)	9	14(100.00)
Aortic sinus of Valsalva	24(6.33)	5(0)	19	19(79.17)
LVOT	5(1.32)	1(0)	4	5(100.00)
LV septum	48(12.67)	25(25)	23	43(89.58)
Anterosuperior septum	12(3.17)	1(1)	11	10(83.33)
Posteroinferior septum	36(9.50)	24(24)	12	33(91.67)
Mitral annulus	5(1.32)	2(1)	3	5(100.00)
LV epicardium	7(1.85)	4(2)	3	4(57.14)
Others(RVIT 3,LV Free wall 3)	6(1.58)	1(0)	5	5(83.33)
Total	379(100.00)	76(39)	303	351(92.61)

VT, ventricular tachycardia; SVT, sustained ventricular tachycardia; RVOT or LVOT, the right or left ventricular outflow tract, respectively; PA, pulmonary artery; PVCs, premature ventricular complexes; LV, left ventricular; RVIT, right ventricular inflow tract; LV epicardium, Idiopathic ventricular arrhythmias that could not be ablated with RFCA from the left sinus of Valsalva despite earliest ventricular activation being recorded in the left sinus of Valsalva or that could be ablated within coronary venous system were classified as originating from the LV epicardium in the present study.

**Table 2:** Baseline characteristics of patients with PVCs/IVT originating from the Tricuspid annulus

Patient	Sex	Age (years)	PVCs/IVT	AADs used	PVC count (No /24 h)	LVEF	Mapping technique	EAT (ms)
1	M	18	PVCs	beta-blocker,Propafenone	21862	62	pace	NA
2	M	18	PVCs	beta-blocker,Propafenone, Mexiletine	19862	69	Pace	NA
3	F	46	PVCs	beta-blocker,Propafenone, Mexiletine	30237	65	EAT + Pace	33
4	M	19	PVCs	beta-blocker,Propafenone, Mexiletine	16804	69	Pace	NA
5	M	67	PVCs	beta-blocker,Propafenone, Mexiletine	16237	62	EAT + Pace	34
6	M	57	PVCs	beta-blocker,Propafenone	22356	51	EAT + Pace	32
7	F	32	PVCs	beta-blocker,Propafenone, Mexiletine	33672	68	EAT + Pace	41
8	F	22	PVCs/NSVT	beta-blocker,Propafenone, Mexiletine	28136	70	EAT + Pace	33
9	F	19	PVCs	beta-blocker,Propafenone, Mexiletine	37691	69	EAT + Pace	32
10	M	32	PVCs	beta-blocker,Propafenone, Mexiletine,Amiodarone	24686	56	Pace	NA
11	M	21	PVCs	beta-blocker,Propafenone, Mexiletine	10086	67	EAT + Pace	30
12	M	14	PVCs	beta-blocker,Propafenone, Mexiletine	36338	68	EAT + Pace	30
13	M	60	PVCs	beta-blocker,Propafenone, Mexiletine	16401	58	EAT + Pace	41
14	F	25	PVCs	beta-blocker,Propafenone	11027	67	EAT + Pace	31
15	M	64	PVCs/NSVT	beta-blocker,Propafenone, Mexiletine,Amiodarone	18511	56	EAT + Pace	35
16	F	23	PVCs	beta-blocker,Propafenone, Mexiletine	18565	71	EAT + Pace	28
17	M	32	PVCs	beta-blocker,Propafenone, Mexiletine	22320	62	EAT + Pace	28
18	M	17	PVCs/NSVT	beta-blocker,Propafenone, Mexiletine	31412	68	EAT + Pace	35
19	M	63	PVCs	beta-blocker,Propafenone	21127	65	EAT + Pace	30
20	M	69	SVT/PVCs	beta-blocker,Propafenone,Amiodarone	19863	52	EAT + Pace	30
21	M	58	SVT/PVCs	Propafenone	20051	61	Pace	NA
22	F	24	PVCs	beta-blocker,Propafenone	21074	68	EAT + Pace	32
23	M	70	PVCs	beta-blocker,Propafenone	17684	62	EAT + Pace	36
24	F	66	PVCs	beta-blocker,Propafenone, Mexiletine	18280	66	EAT + Pace	28
25	M	33	PVCs	beta-blocker,Propafenone	23873	65	EAT + Pace	33
26	F	14	PVCs	beta-blocker,Propafenone	27693	52	EAT + Pace	31
27	M	19	PVCs	beta-blocker,Propafenone,Amiodarone	24132	71	EAT + Pace	31
28	F	50	PVCs	beta-blocker,Propafenone,Amiodarone	17892	54	EAT + Pace	32
29	M	49	PVCs	beta-blocker, Amiodarone	10873	63	Pace	NA
30	F	17	PVCs	beta-blocker,Mexiletine	36817	68	EAT + Pace	30
31	F	23	PVCs	beta-blocker,Propafenone	16382	62	EAT + Pace	32
32	M	58	PVCs	beta-blocker,Propafenone,Amiodarone	10005	64	Pace	NA
33	M	17	PVCs	beta-blocker,Propafenone	13761	71	EAT + Pace	41
34	F	59	PVCs	beta-blocker,Mexiletine	17219	56	EAT + Pace	24
35	M	17	PVCs	beta-blocker,Propafenone	20174	68	EAT + Pace	31
mean ± SD		36.91 ±19.98			21517 ±7429	63.6 ±5.91		32.29 ± 3.93

AADs = antiarrhythmic drugs, LVEF = left ventricular ejection fraction, EAT, the earliest ventricular activation time recorded at ablation target sites that preceded the onset of the QRS complex.

### Baseline 24-hours ECG monitoring (Table 2)

The mean PVC burden during the preoperative 24 hours ambulatory Holter monitoring was 21517 ± 7429 (range 10005 ~ 37691): 16 patients (45.7%) had isolated PVCs, 14 patients(40.0%) had ventricular couplets, 5 patients (14.3%) had monomorphic VT defined as ≥3 consecutive beats of ventricular ectopic beats( 3 nonsustained VT and 2 sustained VT) (Table 2).

**Figure 2:**
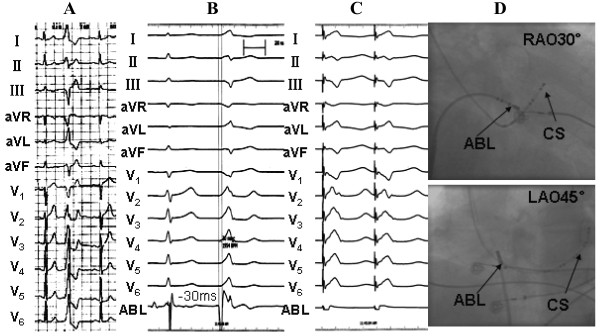
**Recordings obtained at the ablation site for patient number 19 in the Table 2.** (**A**) 12-lead ECG characteristic. (**B**) The local ventricular activation time recorded at the successful ablation site that preceded the onset of the QRS complex was 30 ms. (**C**) Pace map at the successful ablation site. (**D**) The fluoroscopic position of the successful ablation site. ABL, ablation catheter; CS, coronary sinus; RAO, right anterior oblique projection; LAO, left anterior oblique projection.

**Figure 3:**
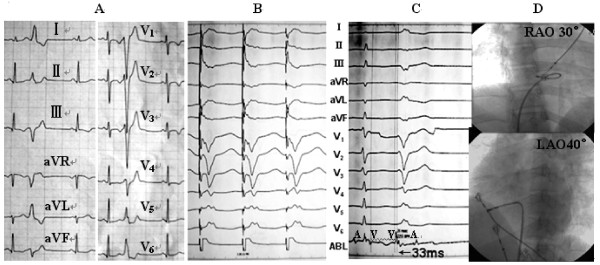
**Recordings obtained at the ablation site for patient number 3 in the Table 2.** (**A**) **12-lead ECG characteristic.** (**B**) Pace map at the successful ablation site. (**C**)The local ventricular activation time recorded at the successful ablation site that preceded the onset of the QRS complex was 33 ms. (**D**) The fluoroscopic position of the successful ablation site. ABL, ablation catheter; RAO, right anterior oblique projection; LAO, left anterior oblique projection.

**Figure 4:**
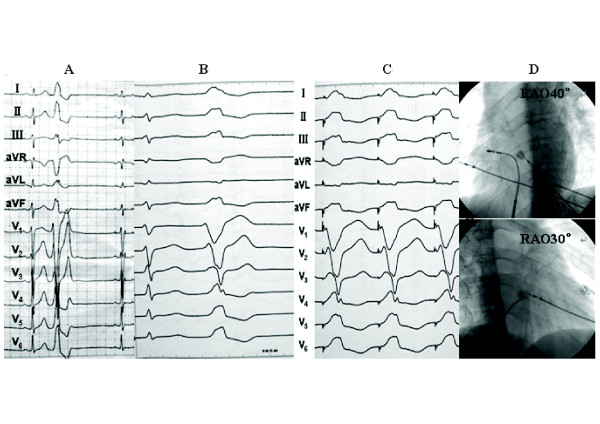
**Recordings obtained at the ablation site for patient number 4 in the Table 2.** (**A**) **12-lead ECG characteristic at a paper speed of 25 mm/s.** (**B**) 12-lead ECG characteristic at a paper speed of 100 mm/s. (**C**) Pace map at the successful ablation site. (**D**) The fluoroscopic position of the successful ablation site. ABL, ablation catheter; RAO, right anterior oblique projection; LAO, left anterior oblique projection

**Figure 5:**
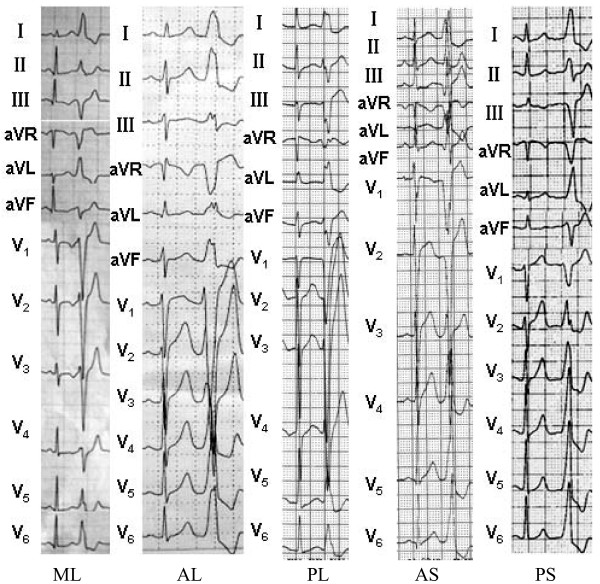
Representative 12-lead ECG characteristics of ventricular arrhythmia originating from Tricuspid annulus.

**Figure 6:**
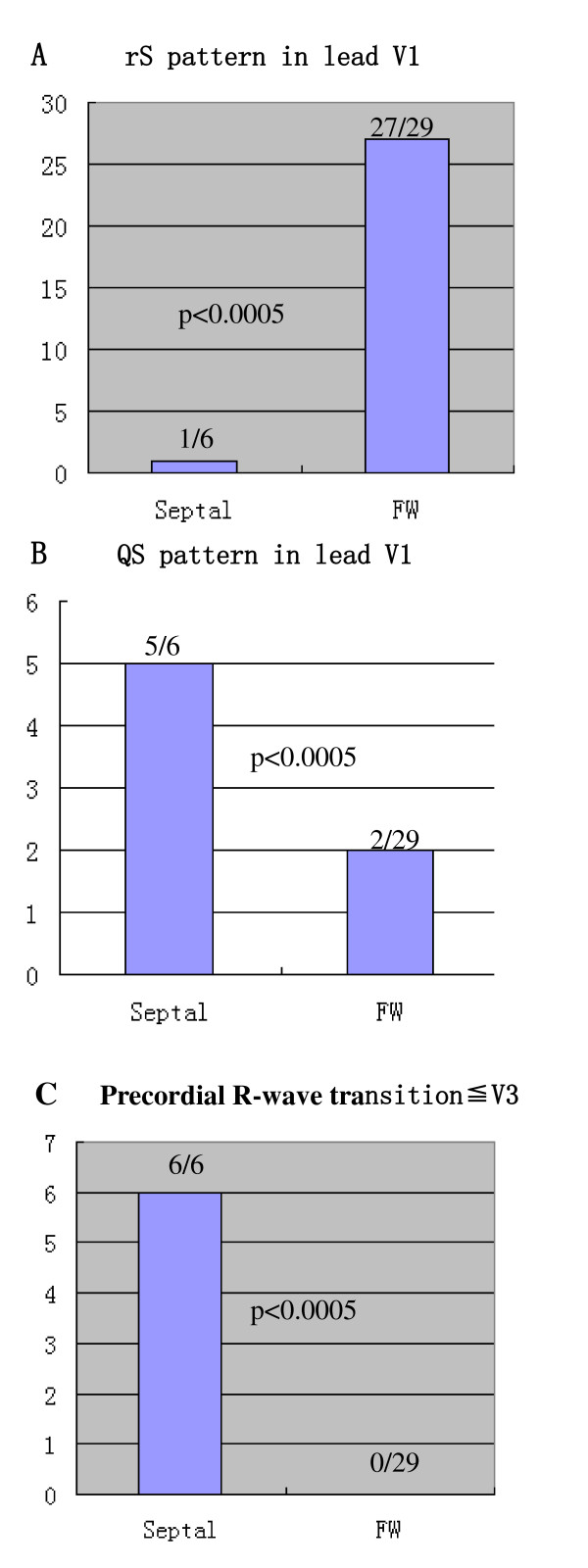
Differences in (A) rS pattern in lead V1, (B) QS pattern inlead V1, and (C) precordial R-wave transition occurring by lead V3 between PVCs/IVTs originating from the septal portion of the tricuspid annulus (Septal origin) and those originating from the free-wall portion of tricuspid annulus (FW origin).

1) Electrophysiologic findings and effect of RFCAPVCs/IVTs in 35 patients originated from the vicinity of TA, including 29 from free wall (10 from anterolateral portion, 12 from midlateral portion, 7 from posterolateral portion), and 6 from septal portion (3 from anteroseptum, 1from midseptum, 2 from posteroseptum). Table 3 lists the detailed VAs origin within the vicinity of TA. The PVCs/IVTs occurred spontaneously in 22 patients and was induced by bolus injection of isoproterenol (2 μg) in 6 patients during the electrophysiologic study. The clinical arrhythmia in the remaining 7 patients could not be induced during the procedure. Sustained VT was not inducible by electrical stimulation and isoproterenol infusion in any patient. All 35 patients underwent the electrophysiologic study using conventional mapping techniques and catheter ablation with temperature-controlled catheter for the VAs. Among patients undergoing activation mapping, the local ventricular activation time preceded the onset of the QRS by 32.29 ± 3.93 ms at successful ablation sites (Table 2).Among the 35 patients with PVCs/IVTs arising from the vicinity of TA, complete elimination of PVCs/IVTs could be achieved by RFCA in 32 patients (success rate 91.43%), including 4 patients with PVCs/IVTs originating from the septal TA, and 28 patients with PVCs/IVTs originating from the free wall portion of the TA (Table 1, Figures 2, 3, 4). Procedure duration (from puncture to removal of sheath catheter ) and radiation exposure time were 75.49 ± 21.34(range 42 ~ 180)min and 11.66 ± 6.17 (range 4.2 ~ 22.7) min, respectively. Thirty-one patients were ablated using a single-catheter approach, and 4 patients using an additional two quadripolar electrode catheters as described. By using pace mapping and activation mapping technique, RFCA was applied in 28 patients, 25 treatments of which were successful, 3 failed (1 originating from the posterolateral portion of TA,7 o’clock position; 2 originating from the anteroseptum of TA,1 o’clock position near the His bundle). PVCs were infrequent despite isoproterenol in the remaining 7 patients, so only pace mapping was performed. All pace mappings were perfect (≧11/12). All the 7 patients were successfully ablated by pace mapping. There were no peri-procedural complications in the 35 patients. Patients have been followed-up for a median of 21 (range 5 ~ 48) months without antiarrhythmic medications. No patient had recurrent ventricular arrhythmia after RFCA.

2) 12-lead ECG characteristics of PVCs/IVTs originating from the vicinity of TAIn all patients with PVCs/IVTs originating from the vicinity of TA, their QRS complex morphology during the PVCs/IVTs showed a left bundle branch block pattern (Figure 5). The duration of the QRS complex of the PVCs/IVTs was 158 ± 15 ms (Table 3). Among the PVCs/IVTs arising from the vicinity of TA, the PVCs/IVTs arising from the septal portion of the annulus had a shorter QRS duration than the PVCs/IVTs arising from the free-wall portion of the annulus (140 ± 14 ms versus 162 ± 13 ms; t = 3.696, P < 0.01). An monophasic R or r pattern in leads I, V5, V6, were recorded in all patients with PVCs/IVTs originating from the vicinity of TA. A positive component (any r or R) was recorded in lead aVL in almost all patients (97.14%), an qs pattern in lead aVL was found in only 1 patient with PVCs/IVTs originating from the vicinity of TA (Table 3). An rS pattern was recorded in lead V1 in 93.1% of patients with PVCs/IVTs from the free wall of TA, vs 16.7% of patients with PVCs/IVTs from the septal TA (p < 0.0005; Figure 6; Sensitivity, specificity, negative predictive value and positive predictive value in Table 4), whereas a QS pattern in lead V1 occurred in 83.3% of patients with PVCs/IVTs from the septal TA vs 6.9% of patients with PVCs from the free wall of the TA (p < 0.0005; Figure 6; Sensitivity, specificity, negative predictive value and positive predictive value in Table 4). The precordial R wave transition occurred by lead V3 or earlier in all patients with PVCs/IVTs originating from the septal portion of the TA, as compared to transition beyond V3 in all patients with PVCs/IVTs from the free wall of the TA (p < 0.0005; Figure 6; Sensitivity, specificity, negative predictive value and positive predictive value in Table 4).

3) Comparison of ECG characteristics of PVCs/IVTs originating from the different portions of the free wall of TAUnlike limb leads, precordial lead QRS morphologies did not differentiate PVCs/IVTs arising from the three portions of the free wall of TA (anterolateral portion, midlateral portion, and posterolateral portion). The following serial changes were noted in limb lead QRS morphologies when the origin of the PVCs /IVTs shifted from posterolateral to midlateral to anterolateral free wall of the TA (Table 5): R wave amplitude increased, and S wave amplitude decreased in leads II, III, aVF; R wave amplitude decreased in leads I, aVL; QS amplitude increased inlead aVR. The amplitude of r or R was usually largest in lead II, next largest in lead aVF, and smallest in lead III (II > aVF > III),and the amplitude of s or S was usually largest in lead III, next largest in lead aVF, and smallest in lead II (III > aVF > II). Among the PVCs/IVTs arising from the free wall portion of TA, Bivariate correlations analysis fit a straight line to the positive relationship between the amplitude of r or R in inferior leads(II, III and aVF) and clock’s position of the origin of PVCs/IVTs in LAO ( from 6 to 12 o’clock) (Table 6); negative relationship between the amplitude of s or S in inferior leads(II, III and aVF) and clock’s position (from 6 to 12 o’clock) . Cut-off values for a R or r-wave amplitude in lead II≦0.20 mv, lead III≦0.15 mv, and lead aVF≦0.175 mv, and for a S or s-wave amplitude in lead II≧0.25 mv and lead aVF≧0.625 mv allowed us to identify all patients with PVCs/IVTs arising from posterolateral free wall of the TA (Table 7).

**Table 3:** 12-lead ECG characteristics of PVCs/IVTs originating from the tricuspid annulus

Pt	PVCs/ VT origin	QRS complex morphology								Transition Zone	QRS duration
		I	II	III	aVR	aVL	aVF	V1	V5~V6		
1	AL	R	R	rs	QS	rsr’	Rs	rS	R	V4	0.16
2	AL	R	Rs	rS	QS	R	RS	rS	R	V4	0.16
3	AL	R	R	rs	QS	rsr’	Rs	rS	R	V4	0.16
4	AL	R	R	Rs	qs	rsr’	R	rS	R	V3	0.15
5	AL	r	Rs	rs	QS	r	Rs	rS	R	V4	0.16
6	AL	R	r	r	QS	r	r	rS	R	V3	0.16
7	AL	R	Rs	rS	QS	R	RS	rS	R	V4	0.20
8	AL	R	Rs	Rs	QS	R	Rs	rS	R	V4	0.16
9	AL	r	Rs	Rs	QS	qs	Rs	rS	R	V4	0.16
10	AL	R	Rs	RS	QS	r	Rs	rS	R	V3~V4	0.17
11	ML	R	rs	rS	qs	qR	rS	rS	R	V4~V5	0.14
12	ML	R	Rs	rs	QS	R	rs	rS	R	V4~V5	0.18
13	ML	R	rs	rS	QS	R	rS	QS	R	V4~V5	0.14
14	ML	R	rs	rS	qs	R	rs	rS	R	V4	0.15
15	ML	R	rs	rS	qs	R	rs	rS	R	V4~V5	0.14
16	ML	R	rs	rS	QS	R	rS	rS	R	V4	0.17
17	ML	R	rs	rS	QS	R	rS	rS	R	V3~V4	0.16
18	ML	R	rs	rS	QS	R	rS	rS	R	V3~V4	0.17
19	ML	R	rs	rS	QS	R	rS	rS	R	V3~V4	0.17
20	ML	R	rs	rS	qs	R	rs	rS	R	V4~V5	0.16
21	ML	R	Rs	rS	QS	R	rs	rS	R	V4	0.18
22	ML	R	rs	rs	qs	R	rs	rS	R	V4	0.16
23	PL	R	rs	rS	QS	R	rS	rS	R	V4	0.16
24	PL	R	rS	rS	QS	R	rS	rS	R	V3	0.16
25	PL	R	rs	rS	qs	R	rS	rS	R	V3	0.15
26	PL	R	rsr’	rS	qs	R	rS	rS	R	V3	0.17
27	PL	R	rS	rS	QS	R	rS	QS	R	V5	0.15
28	PL	R	rs	rS	QS	R	rS	rS	R	V3	0.16
29	PL	R	rs	rS	qs	R	rS	rS	R	V4	0.18
30	AS	R	R	RS	QS	R	R	QS	R	V2	0.16
31	AS	R	R	rsr’	QS	R	R	QS	R	V2	0.14
32	AS	R	R	qRs	QS	R	qRs	QS	R	V2~V3	0.15
33	MS	R	rs	rS	QS	R	rs	QS	R	V3	0.13
34	PS	R	rS	rS	qr	R	rS	rS	R	V3	0.12
35	PS	R	rs	rS	QS	R	rS	QS	R	V1~V2	0.14

AL,anterolateral portion; ML, midlateral portion; PL, posterolateral portion; AS, anteroseptum; MS, midseptum; PS, posteroseptum.

**Table 4:** The sensitivity, specificity, negative predictive value (NPV) and positive predictive value (PPV) to identify the precise origin of PVCs/IVTs from the tricuspid annulus (TA)

ECG variables	Sensitivity (%)	Specificity (%)	NPV (%)	PPV (%)
rS pattern in lead V1 in patients with PVCs/IVTs arising from the free wall of TA	93.10	83.33	96.43	71.43
Precordial R-wave transition ≤ V3 in patients with PVCs/IVTs arising from the septal TA	100	100	100	100
QS pattern in lead V1 in patients with PVCs/IVTs arising from the septal TA	83.33	93.10	71.43	96.43

**Table 5:** Comparision of the amplitude of R(r) or S (s) or QS in limb leads

Group	n	R_I_	II		III		QS_aVR_	R_aVL_	aVF	
			r(R)	s(S)	r(R)	s(S)			r(R)	s(S)
PL	7	1.02 ± 0.44	0.09 ± 0.02	−0.50 ± 0.24	0.05 ± 0.03	−1.48 ± 0.67	−0.44 ± 0.18	1.13 ± 0.53	0.08 ± 0.02	−0.98 ± 0.51
ML	12	0.87 ± 0.21	0.33 ± 0.12^e^	−0.24 ± 0.17^e^	0.17 ± 0.09^e^	−0.83 ± 0.35^e^	−0.47 ± 0.12	0.83 ± 0.26^e^	0.22 ± 0.09^e^	−0.50 ± 0.22^e^
AL	10	0.58 ± 0.17^ac^	0.78 ± 0.28^ac^	−0.08 ± 0.14^bc^	0.38 ± 0.17^b c^	−0.31 ± 0.25 ^ac^	−0.69 ± 0.18^b^	0.37 ± 0.32^a c^	0.58 ± 0.21^a c^	−0.21 ± 0.19 ^a c^

AL, anterolateral portion; ML, midlateral portion; PL, posterolateral portion;

^a^p < 0.01,^b^p < 0.05, AL vs.ML; ^c^p<0.01^,d^p<0.05, AL vs.PL ;^e^p<0.01,^f^p<0.05, MLvs.PL.

**Table 6:** The relationship between the amplitude of r(R) or s(S) or QS in12-lead ECG and clock’s position of the origin of PVC/VT

Leads	Amplitude of r(R)		Amplitude of s(S) or QS	
	*r* value	*p* value	*r* value	*p* value
I	−0.4565	<0.02	/	/
II	0.8131	<0.001	−0.5301	<0.005
III	0.7264	<0.001	−0.8328	<0.001
aVR	/	/	−0.5590	<0.01
aVL	−0.6601	<0.001	/	/
aVF	0.8128	<0.001	−0.7405	<0.001
V_1_	−0.0459	>0.05	0.1178	>0.05
V_2_	−0.1120	>0.05	0.1451	>0.05
V_3_	−0.2526	>0.05	0.1193	>0.05
V_4_	0.0840	>0.05	0.2819	>0.05
V_5_	0.1418	>0.05	/	/
V_6_	0.3955	<0.05	/	/

Bivariate correlations analysis fit a straight line to the positive relationship between the amplitude of r or R in inferior leads(II, III and aVF) and clock’s position of the origin of PVCs/IVTs in LAO ( from 6 to 12 o’clock); negative relationship between the amplitude of s or S in inferior leads (II, III and aVF) and clock’s position (from 6 to 12 o’clock).

**Table 7:** The cut-off values to identify PVCs/IVTs from posterolateral free wall of the TA

**Leads**	Cut-off values*	**Sensitivity(%)**	**Specificity(%)**
r(R)II	0.200 mv	100	100
s(S)II	−0.250 mv	100	100
r(R)III	0.150 mv	100	100
s(S)III	−0.975 mv	85.7	100
r(R)aVF	0.175 mv	100	100
s(S)aVF	−0.625 mv	100	100

*≦Cut-Off vaules for the r(R) or s(S) wave amplitude in inferior leads in patients with PVCs/IVTs arising from posterolateral free wall of the TA.

## Discussion

Two major findings were obtained in the present study. First, 9.23% of PVCs/IVTs had an origin in the vicinity of TA, and PVCs/IVTs arising from the vicinity of TA, especially the free wall portion of TA, were safely and effectively eliminated with RFCA. Second, PVCs/IVTs originating from the vicinity of TA had distinctive ECG characteristics that were useful for identifying the precise origin. In all patients with PVCs/IVTs originating from the vicinity of TA, the QRS complex morphology during the PVCs/IVTs showed a left bundle branch block pattern. No negative component of the QRS complex was found in lead I, V5,V6. An early precordial R-wave transition by lead V3 and an QS pattern in lead V1 were useful for differentiating the origin of PVCs/IVTs from the free wall portion and septal portion of TA. Among the PVCs/IVTs arising from the free wall portion of TA, the amplitude of r or R in the inferior leads(II, III and aVF) was straight-line associated with the clock’s position of the origin of PVCs/IVTs in LAO ( from 6 to 12 o’clock).

In this study, we demonstrated that RFCA was effective for eliminating PVCs/IVTs arising from the vicinity of TA. On the basis of conventional mapping techniques (pace mapping technique and/or activation mapping), 32 (91.43%) of 35 patients with PVCs/IVTs arising from the vicinity of TA were successfully ablated. No patient had recurrent ventricular arrhythmia after acute successful RFCA during a median follow-up period of 21 months. No significant complications were observed in the present study confirming the safety of the procedure. For PVCs/IVTs arising from the free wall portion of TA, the ablation success rate was 96.55% (28 of 29 patients). However, the success rate was only 66.67% (4 of 6 patients) for PVCs/IVTs arising from the septal portion of the TA. Thus, RF catheter ablation was more effective for PVCs/IVTs arising from the free wall portion of TA than that from arising the septal portion of the TA. The results of this study was similar to a previous study [13]. However, the total success rate (91.43%) in the present study was higher than that in the previous study (66%). The reason was associated with the origin of PVCs/IVTs. In the present study, 29 PVCs/IVTs (82.86%) in all 35 patients originated from the free wall portion of TA, PVCs/IVTs originated from the septal portion in only 6 patients. In the previous study [13], 28 PVCs/IVTs (74%) originated from the septal portion of the TA and the remaining 10 (26%) from the free wall of the TA. The origin of PVCs/IVTs in these two studies resulted in the different ablation success rate. The reason for the significantly different preferential site of origin in both the present and previous study (the preferential site of origin was the free wall of TA in our study, but the septal portion of TA in the previous study) is not clear, and might be associated with environment or region. In addition, 31 of 35 patients were ablated using a single-catheter approach in the present study. Thirty of 31 patients ablated using a single-catheter approach originated from the free wall portion of TA. All of them were ablated successfully. Thus, the single-catheter approach to radiofrequency ablation for PVCs/IVTs arising from the free wall portion of TA, is feasible, safe, effective.

In this study, we found that PVCs/IVTs originating from the vicinity of TA have distinctive ECG characteristics. The most interesting finding was that when the origin of PVCs/IVTs shifted from posterolateral portion to midlateral portion to anterolateral portion of TA, R wave amplitude were more increasing and S wave amplitude was reduced even disappearing in the inferior leads(II, III and aVF), R wave amplitude were more lessening in leads I and aVL, QS wave amplitude were more increasing in lead aVR, and the amplitude of r or R in the inferior leads was straight-line associated with clock’s position of the origin in LAO ( from 6 to 12 o’clock) among the PVCs/IVTs arising from the free wall portion of TA. Movement of the origin of PVCs/IVTs from an inferolateral to anterolateral TA led to greater R wave in inferior leads. Therefore, more large clock’ position, more greater R or r wave in the inferior leads. For example, the amplitude of R or r wave for PVCs/IVTs located at 7 o’clock position was less than that at 11 o’clock position. Because the origin of the PVCs/IVTs arising from the posterolateral portion of TA (6–8 o’clock position) was located on the right inferior side of the heart, the myocardium would be depolarized in a direction toward the anode of lead aVL, away from the inferior leads, and vertical toward aVR, which might account for the findings: negative QRS polarity in the inferior leads (S_III_ > S_aVF_ > S_II_,r_II_ > r_aVF_ > r_III_), positive QRS polarity in lead aVL; the origin of the PVCs/IVTs arising from the anterolateral portion of TA (10–12 o’clock position) was located on the right superior side of the heart, the myocardium would be depolarized in a direction toward the anode of lead II, away from the lead aVR, which might account for the findings: positive QRS polarity in the inferior leads (R_II_ > R_aVF_ > R_III_,s_III_ > s_aVF_ > s_II_), negative QRS polarity in lead aVR.

### Study limitations

First, the mechanism of the PVCs/IVTs arising from the vicinity of the TA remains speculative in this study. In the present study, programmed electrical stimulation could not induce sustained ventricular tachycardia in any patient. The QS unipolar morphology would be a strong pointer towards focal automaticity. However, the unipolar lectrogram was not recorded at the site of earliest activation in the present study. Second, no abnormalities suggestive of arrhythmogenic right ventricular cardiomyopathy were found by ECG and echocardiography and right ventricular contrast angiography in any of the patients with the TA ventricular arrhythmias in the present study. However, a signal-averaged ECG or endomyocardial biopsy or MRI or electroanatomic mapping in any of the patients was no performed. Therefore, we could not have completely excluded the possibility of a concealed form of arrhythmogenic right ventricular cardiomyopathy. However, we believe the data presented strongly suggests that the arrhythmias described were truly idiopathic. To increase the accuracy of our study, our results need to be confirmed in additional long-term follow-up.

## Conclusions

RFCA is an effective curative therapy for symptomatic PVCs/IVTs originating from the vicinity of TA. There are specific characteristics in ECG and the ablation site could be located by ECG analysis.

## Competing interests

The authors declare that they have no competing interests.

## Authors’ contributions

LJF and LYC designed the whole study, LJF, LYC, ZWW, ZND, ZT, WPX, GB, LJ and JKT performed the experiment, LJF and LYC wrote the paper. All authors read and approved the final manuscript.

## Pre-publication history

The pre-publication history for this paper can be accessed here:

http://www.biomedcentral.com/1471-2261/12/32/prepub
